# SCAPS simulation of novel inorganic ZrS_2_/CuO heterojunction solar cells

**DOI:** 10.1038/s41598-023-31553-4

**Published:** 2023-03-20

**Authors:** Mahmoud Abdelfatah, Adel M. El Sayed, Walid Ismail, Stephan Ulrich, Volker Sittinger, Abdelhamid El-Shaer

**Affiliations:** 1 Physics Department, Faculty of Science, KafrelSheikh University, KafrelSheikh, 33516 Egypt; 2 Physics Department, Faculty of Science, Fayoum University, Fayoum, 63514 Egypt; 3 Fraunhofer Institute for Surface Engineering and Thin Films IST, Bienroder Weg 54E, 38108 Braunschweig, Germany

**Keywords:** Materials science, Nanoscience and technology, Physics

## Abstract

ZrS_2_ is transition metal dichalcogenides (TMDCs) which is believed one of the most talented applicants to fabricate photovoltaics. Therefore, we present here for the first-time numerical simulation of novel inorganic ZrS_2_/CuO heterojunction solar cells employing SCAPS-1D. The influence of the thickness, carrier concentration, and bandgap for both the window and absorber layers on the solar cell fundamental parameters was explored intensely. Our results reveal that the solar cell devices performance is mainly affected by many parameters such as the depletion width (**W**_**d**_), built-in voltage (**V**_**bi**_), collection length of charge carrier, the minority carrier lifetime, photogenerated current, and recombination rate. The **η** of 23.8% was achieved as the highest value for our simulated devices with the **V**_**oc**_ value of 0.96 V, the **J**_**sc**_ value of 34.2 mA/cm^2^, and the **FF** value of 72.2%. Such efficiency was obtained when the CuO band gap, thickness, and carrier concentration were 1.35 eV, 5.5 µm, and above 10^18^ cm^−3^, respectively, and for the ZrS_2_ were 1.4 eV, 1 µm, and less than 10^20^ cm^−3^, respectively. Our simulated results indicate that the inorganic ZrS_2_/CuO heterojunction solar cells are promising to fabricate low-cost, large-scale, and high-efficiency photovoltaic devices.

## Introduction

Currently, the demand for energy increase year by year, which is important for technological and industrial development worldwide although limited fossil fuels^1^. Solar cells are considered one of the highly significant renewable energy sources since they are environmental ecofriendly technology to reduce global CO_2_ emissions^1^.

Recently, transition metal dichalcogenides (TMDCs) have received high attention to become potential candidates instead of traditional materials for various applications especially solar cells^2,3^ as well as batteries^4^, photodetector^5,6^, biomedical^7^, and catalysis^8^. In these materials, the d- d transition positioned at a metal site producing large band-edge excitation and therefore unique electronic properties leading for such various applications.

Zirconium disulfide (ZrS_2_), belongs to group IV of TMDCs, is n-type semiconductor which reveals a low mismatch lattice with other absorber materials because of Van-der-Waals force (VdW) force^2,9^. ZrS_2_ is considered as a powerful applicant to fabricate optoelectronics particularly photovoltaics since it has high absorption coefficient and bandgap energy that could be easily engineered to be in the range of 1.2–2.2 eV^2,3,10^. Moreover, ZrS_2_ has several unique electronic and optical properties owing to quasi 2D characteristic^2^. Based on these properties, ZrS_2_ could be utilized in many fields besides solar cells^2,3^ such as field-effect transistors (FETs)^11^, lithium-ion batteries^12^, water-splitting^13^, photocatalysis^14^, lubricant additives^15^, and photodetectors^16^.

Tunable of bandgap energy as well as electronic and optical properties for ZrS_2_ thin film could be achieved employing several growth techniques like chemical vapor deposition (CVD)^11^, chemical vapor transport (CVT)^17^, atomic layer deposition (ALD)^13,18^, sputtering^19^, and liquid exfoliation method^9^.

On the other hand, CuO is a semiconductor that has bandgap in the range of 1.2–1.5 eV, good thermal and electronic features and generally used in superconductors, supercapacitors, and solar energy purposes^20^. In addition, Cu_2_O is a non-toxic material and has a narrow direct optical band gap in the range of 1.9–2.3 eV and therefore employed on optoelectronic devices^21^. The combination of *n*-ZrS_2_ thin films with other *p*-type semiconductors with appropriate energy level alignment such as CuO and Cu_2_O as well as buffer layers like graphene oxide could be future key to have solar cells with higher efficiency^22–25^.

The predication of the optimum parameters for the materials is considered one the most promising keys to fabricate high performance solar cells. The simulation is a valuable approach to examine the influence of changes in material properties on the solar cell basic parameters and study the complex heterogeneous absorber/buffer interfaces. Therefore, many papers were published to predicate the solar cells efficiencies employing Solar Cell Capacitance Simulator Structures (SCAPS-1D) software, which is a one-dimensional (1D) windows-oriented program and has the largest number of simulation parameters^26–29^.

Consequently, we present here for the first-time SCAPS numerical simulation of novel inorganic ZrS_2_/CuO heterojunction solar cell devices. Typically, the effect of the thickness, carrier concentration, and bandgap for both the window and absorber layers on the solar cell fundamental parameters will be investigated deeply. Our simulated results will be step forwards to know the optimum parameters to experimental produce high efficiency photovoltaic devices.

## Materials and methods

### Inorganic solar cell structure

**Figure 1:**
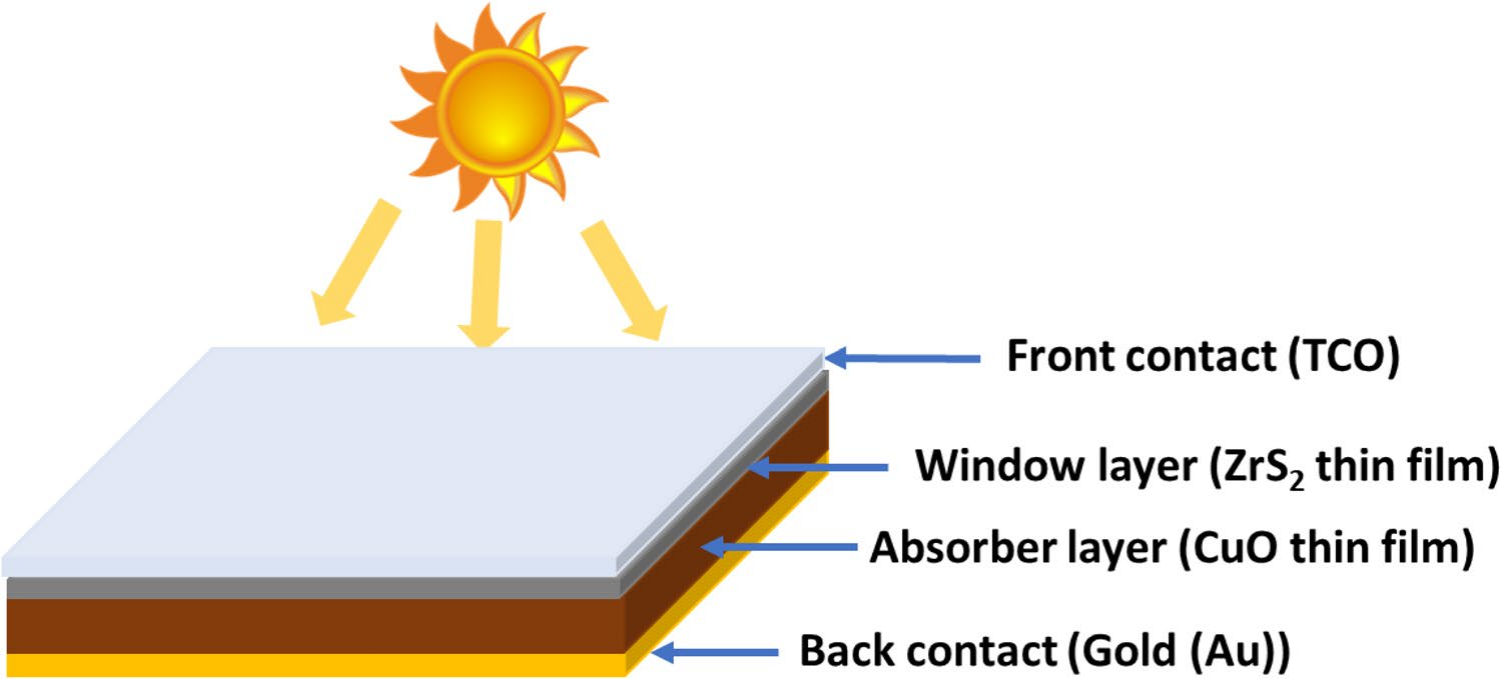
Schematic structure of inorganic ZrS_2_/CuO heterojunction solar cell.

Figure 1 represents a schematic diagram of the heterojunction device structure that was employed in the simulation. Typically, n-ZrS_2_ thin film was used as the window layer, while p-CuO thin film was employed as the absorber layer to shape our inorganic p–n heterojunction solar cells. Transparent conductive oxide (TCO) and Gold (Au) were utilized as the front and the back metal contacts for the device.

### Numerical simulation and parameters of materials

In our current research, SCAPS-1D software^30–32^, as a powerful and valuable numerical simulation tool to recognize and clarify the physical phenomena arising in photovoltaic devices, was utilized to simulate and evaluate our inorganic solar cells. The light intensity of AM 1.5 light spectrum (100 mW/cm^2^), as standard testing conditions (STC), were employed in all SCAPS-1D simulation calculations. Here, impact of bandgap, thickness, and carrier concentration of both the absorber and window layers on the solar cell fundamental parameters were analyzed intensely where different values for input variable were used to obtain optimum values to have higher performance of solar cell devices.

The following Poisson and continuity equation for holes and electrons are used in SCAPS-1D numerical simulation calculations^33,34^

1 $$\frac{{d}^{2}}{{dx}^{2}}\Psi \left(x\right)=\frac{e}{{\varepsilon }_{0}{\varepsilon }_{r}} \left(p\left(x\right)-\mathrm{n}\left(\mathrm{x}\right)+{N}_{D}- {N}_{A}+{\rho }_{defect}(\mathrm{p}\left(\mathrm{x}\right),\mathrm{n}\left(\mathrm{x}\right)) \right)$$

2 $$\frac{d{J}_{n}}{dx}= \mathrm{G}-\mathrm{R \,and\, } \frac{d{J}_{p}}{dx}= \mathrm{G}-\mathrm{R}$$

where **Ψ**, **e**, **ε**_**0**_, **ε**_**r**_, **p**, **n**, **N**_**D**_, **N**_**A**_, **ρ**_***defect***_,** J**_**n**_, **J**_**p**_, **R**, and **G** are electrostatic potential, charge of electron, vacuum permittivity, relative permittivity, hole density, electron density, donor impurities, acceptor impurities, distribution of defects, current densities of electron, current densities of hole, recombination rate, and generation rate, respectively. The parameters of ZrS_2_ and CuO thin films applied to execute our numerical simulations are scheduled in Table 1 according to Refs.^2,25,35,36^.

**Table 1:** Material parameters employed in our simulations^2,25,35,36^.

Material parameter	n-ZrS_2_	p-CuO
Dielectric permittivity	16.4	18.1
Bandgap (eV)	1.7	1.2
Electron affinity (eV)	4.7	4.07
Effective density of states of valence band maximum (cm^−3^)	1.8 × 10^19^	5.5 × 10^20^
Effective density of states of conduction band minimum (cm^−3^)	2.2 × 10^19^	3 × 10^19^
Acceptor concentration (cm^−3^)	0	10^16^
Donor concentration(cm^−3^)	10^19^	0
Mobility of Hole (cm^2^/(V · s))	30	20
Mobility of Electron (cm^2^/(V · s))	300	200
Electron thermal velocity (cm/s)	10^7^	10^7^
Hole thermal velocity (cm/s)	10^7^	4.6 × 10^6^

## Results and discussion

### Impact of CuO thickness, bandgap, and carrier concentration on basic parameters of solar cells

In this section, we firstly study effect of the absorber layer thickness, band gap, and carrier concentrations on the photovoltaic fundamental parameters (open circuit voltage (V_oc_), short circuit current density (J_sc_), fill factor (FF), and cell efficiency (η)) where the window layer thickness, band gap, and carrier concentration are fixed at 0.2 μm, 1.7 eV, 10^19^ cm^−3^, respectively.

**Figure 2:**
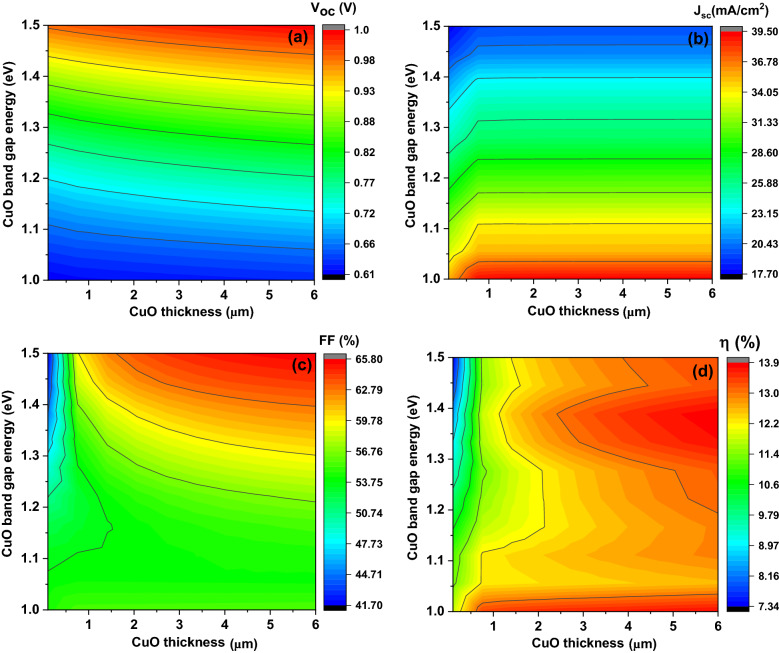
The simulated solar cells basic parameters including V_oc_ (**a**), J_sc_ (**b**), FF (**c**), and η (**d**) as function on the absorber layer thickness (x-axis) and the band gap (y-axis) at acceptor concentration 10^16^ cm^−3^.

Figure 2 demonstrates the gained contour plot of the modelled solar cell basic parameters including V_oc_, J_sc_, FF, and η as the CuO layer thickness and the band gap vary from 1 to 6 μm (the x-axis) and from 1 to 1.5 eV (the y-axis), respectively.

It is clear from Fig. 2a that **V**_**oc**_ increases from about 0.61 V at **E**_**g**_ of 1 eV to about 1 V at **E**_**g**_ of 1.5 eV while the variation of the **V**_**oc**_ with CuO thickness rise from 1 to 6 µm for any Eg did not excess more than 0.1 V. The **J**_**sc**_ value is observed to decline from about 39.5 to 17.7 mA/cm^2^ with the increase of the **E**_**g**_ from 1 to 1.5 eV as presented in Fig. 2b. The **J**_**sc**_ values remain almost constant for each CuO thickness which varied from 1 to 6 µm.

Figure 2c indicates that the FF values are varying in different ways where firstly FF decreases from about 55% to about 41.7% with increasing of the **E**_**g**_ from 1 to 1.5 eV and this at the same time of increasing CuO thickness from about 0.1–1.5 µm (thinner CuO thin films). Secondly, FF grows from about 55% to about 65.8% with raising of the **E**_**g**_ from 1 to 1.5 eV and this take place with CuO thickness increasing from about 1.5–6 µm (thicker CuO thin films) with reducing on the value of FF of about 3%.

Figure 2d reveals that the η values have different zones where η of about 13.3% is detected at the **E**_**g**_ of about 1 eV with thickness increasing from about 0.5 to 6 µm. η with average value of about 12.5% is achieved for almost variation of the **E**_**g**_ from 1 to 1.5 eV expect zone located at the **E**_**g**_ from 1.3 to 1.45 eV and the thickness from 3 to 6 µm where higher performance of about 13.86% is obtained.

These results could be clarified as the band gap increasing, the local collection efficiency of light absorption increase inside the interface of the p-CuO thin film and n-ZrS_2_ thin film and enhances the carrier generation rate and therefore increasing the value of the **V**_**oc**_ dramatically according to the following equation^37^:

3 $${V}_{oc}=\frac{{k}_{B}\mathrm{T }}{\mathrm{q}}\mathrm{ln} \left(1+\frac{{I}_{ph}}{{I}_{0}}\right)$$

Also, this explained whereas based on the ZrS_2_ and CuO bandgap values, band alignment of a cliff-like is supposed leading to improve the charge separation process^2^. When the bandgap energy raises, the valence band **Ev** shifts downward while the conduction band **Ec** shifts upper producing decreasing on the conduction band offset (**ΔEc**) value^2^. The lowered **ΔEc** helps the charge separation process with more approachable promotion electrons to the absorber layer and transport through the interface into the window layer and therefore enhancement of the **V**_**oc**_^2^. While band gap increasing producing high hole concentrations close to the interface junction. Therefore, increasing the probability of the recombination rate happens through surface and facing interface recombination. Consequently, this explained reducing of the **J**_**sc**_ with increasing band gap^2,38^. Moreover, the wavefunction intersecting between vibrational excited states of the lower and the higher lying electronic states produce capture centers for charge carriers^38^.

The behavior of the **FF** is always effected by many factors especially the series and shunt resistance as well as partially the **V**_**oc**_ values as presented in the following equation^39^:

4 $$\mathrm{FF}=\frac{{{\varvec{\upupsilon}}}_{oc}-\mathrm{ln}({{\varvec{\upupsilon}}}_{oc}+0.72)}{{{\varvec{\upupsilon}}}_{oc}+1},$$

where **υ**_**oc**_** = qV**_**oc**_**/AkT**.

The η behavior could be illustrated by the behavior and the values of the **V**_**oc**_, the **J**_**sc**_, and the **FF** where the **η** given by the following equation^40^:

5 $$\upeta =\frac{{FF V}_{oc}{ J}_{sc}}{{ P}_{in}}.$$

**Figure 3:**
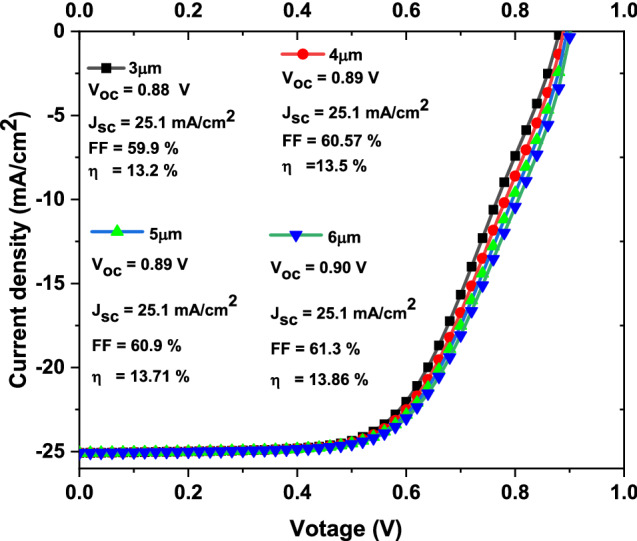
The simulated JV curves with basic parameters of solar cells as function on CuO thickness from 3 to 6 µm.

From above results we concluded that to have higher efficiencies solar cells, the CuO band gap and thickness should be in the range of 1.35 eV and from 3 to 6 µm, respectively. Therefore, JV curves with basic parameters of solar cells as function of CuO thickness from 3 to 6 µm at constant band gap of 1.35 eV are simulated as described in Fig. 3. It is clear from such figure that the **V**_**oc**_ has value from 0.88 to 0.90 V, the **J**_**sc**_ has constant value of 25.1 mA/cm^2^, the **FF** has value from 59.9 to 61.3% and the **η** has value from 13.2 to 13.86 with increasing thickness from 3 to 6 µm, respectively where the **η** value follows mainly the **V**_**oc**_, and the **FF** values.

**Figure 4:**
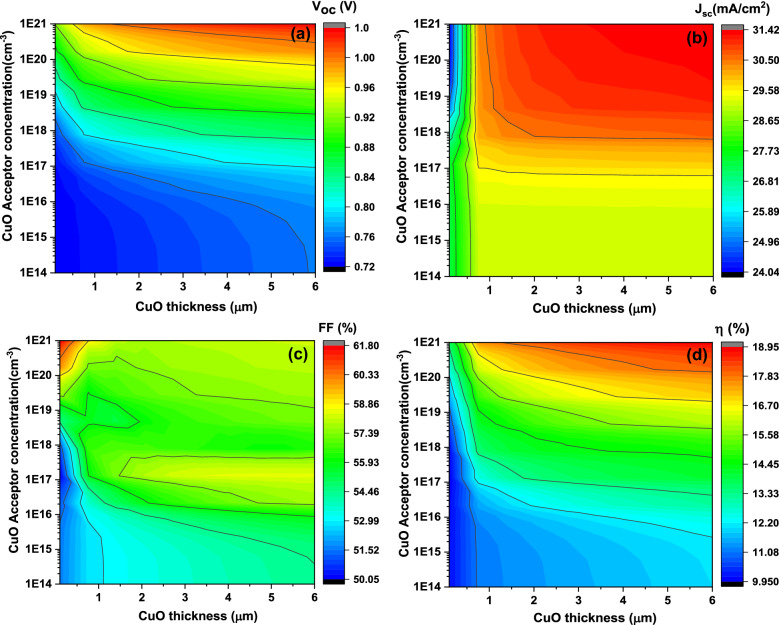
The simulated solar cells basic parameters including V_oc_ (**a**), J_sc_ (**b**), FF (**c**), and η (**d**) as function on the absorber layer thickness (x-axis) and the carrier concentration (y-axis) at E_g_ = 1.35 eV.

Figure 4 displays the achieved contour plot of the obtained photovoltaic basic parameters consist of V_oc_, J_sc_, FF, and η as function on the CuO thin film thickness and the acceptor concentration differ from 1 to 6 μm (the x-axis) and from 1 × 10^14^ to 1 × 10^21^ cm^−3^ (the y-axis), respectively.

It is apparent from Fig. 4a that the **V**_**oc**_ improves from about 0.72 V to 1 eV as acceptor concentration enhances from 10^14^ to 10^21^ cm^−3^ with small variation in the **V**_**oc**_ values with rising of CuO thickness. From Fig. 4b, the value of **J**_**sc**_ increases from about 29.5–31.4 mA/cm^2^ with increasing of acceptor concentration from 10^17^ to 10^21^ cm^−3^ and the value stays constant at about 29.5 mA/cm^2^ for carrier concentration less than 10^17^ and this at CuO thickness above 1.5 µm. For CuO thickness less than 1.5 µm, the **J**_**sc**_ continues of about 28.3 mA/cm^2^ for carrier concentration less than 10^18^ cm^−3^, while the **J**_**sc**_ value change for carrier concentration above 10^18^ cm^−3^ from 24 to 29.5 mA/cm^2^ with CuO thickness increasing.

Figure 4c implies that the FF values remain mainly constant at value of about 56% for all carrier concentration and thickness expect for carrier concentration from 10^14^ to 10^17^ cm^−3^, the FF increase from about 50–55% in thickness range from 1 to 3 5 µm. The FF higher value of about 61% is obtained for carrier concentration of 10^21^ cm^−3^ and for thickness less than 1 µm.

Figure 4d shows that the η values have the same shape as the **V**_**oc**_ where η increase from about 9.8% to about 12.5% as the carrier concentration increase from 10^14^ to 10^17^ cm^−3^ and this with thickness growing. The η value of about 12.5% increase to about 18.9% with rising of the carrier concentration from 10^17^ to 10^21^ cm^−3^.

Such results could be explained as the carrier concentration increasing, the electron diffusion increases from the p-CuO thin film to n-ZrS_2_ thin film and enhances the value of the device built-in voltage (**V**_**bi**_) which formed mainly by the depletion width (**W**_**d**_) and therefore the **V**_**oc**_ increases dramatically^37,38^. Moreover, the photogenerated current (electron hole pairs) (**I**_**ph**_) increase and therefore the recombination rate and the reverse saturation current (**I**_**o**_) will be reduced and therefore enhancement of the **V**_**oc**_ values^41^ as presented in Eq. (3) which clear the relation between the **V**_**oc**_, **I**_**ph,**_ and** I**_**o**_ _^42^:

On other hand, the **J**_**sc**_ values improves with increasing of carrier concentration because also enhancement of the photogenerated charge carriers and decreasing of both the leakage current and the recombination current especially for CuO thickness higher than 1.5 µm.

For the absorber thickness less than 1.5 µm and carrier concentration above 10^18^ cm^−3^, the **J**_**sc**_ raise with CuO thickness growing could be ascribed to the CuO charge collection length and hence the collection of electron hole pairs in the junction will be improved^43^. This indicates that the charge carriers will be split up and stored clearly with thickness increasing. For higher carrier concentration than 10^18^ cm^−3^ and CuO thickness higher than 1.5 µm, the charge collection length reached the maximum and the carrier collection probability increasing and the recombination rate and leakage current reducing which producing higher values for the **J**_**sc**_^2^.

**Figure 5:**
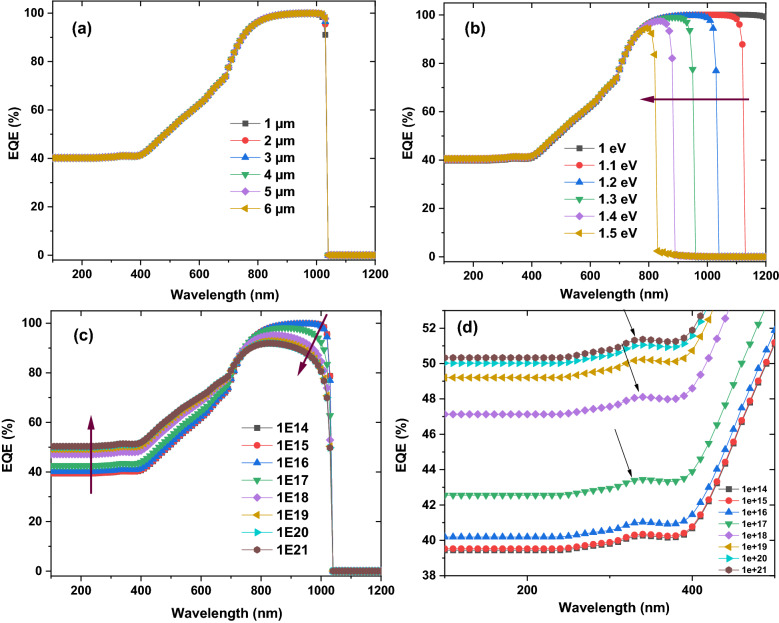
The simulated external quantum efficiency as function on CuO thickness (**a**), band gap (**b**), and the carrier concentration (**c,d**) respectively.

The behavior of the η could be described by the whole behavior of the **V**_**oc**_, the **J**_**sc**_, and the **FF** where the η is a factor of these parameters as shown before in Eq. (5)^40^:

In this case, the shape of the η follows mainly the **J**_**sc**_ shape since the acceptor concentration influence mainly the photogenerated current. Therefore, the external quantum efficiency (EQE) as function on CuO thickness, band gap, and the carrier concentration is recorded as presented in Fig. 5. It is noticeable that EQE have the same shape with all CuO thickness with value of 40% at 200 nm and 100% at 800 nm. With increasing of the band gap from 1 to 1.5 eV, the area under curve for higher EQE part moved downward from 1200 to 800 nm. By increasing the carrier concentration from 10^14^ to 10^21^ cm^−3^, EQE value improve from around 40–52% for wavelength from 200 to 700 nm, while EQE value reduce from 100 to 90% for higher wavelength from 700 to 1050 nm. Moreover, there is intrinsic absorption edge in the short-wavelength region of the spectrum^41,42^ where it positions change with the variation of the carrier concentration as presented in Fig. 5d and this could be attributed to the reflection from the Au back contact^41,42^. The results indicate the photogenerated current increasing with high energy photon while decreasing for lower one and this suppose the different generation and recombination rate of charge carriers that controlled the **J**_**sc**_. The results confirmed and supported that the η will affect mainly by the **J**_**sc**_. We can conclude from the above simulations that the CuO band gap, thickness, and carrier concentration should be in the range of 1.35 eV, 5.5 µm, and above 10^18^ cm^−3^, respectively to achieve higher efficiencies for ZrS_2_/CuO heterojunction solar cell devices.

### Impact of ZrS_2_ thickness, bandgap, and carrier concentration on basic parameters of solar cells

Secondly, the influence of the ZrS_2_ thin film, as window layer, thickness, band gap, and carrier concentrations on the basic parameters of photovoltaic devices will be explored deeply while the CuO layer thickness, band gap, and carrier concentrations are fixed at 5.5 μm, 1.35 eV, 10^18^ cm^−3^, respectively.

**Figure 6:**
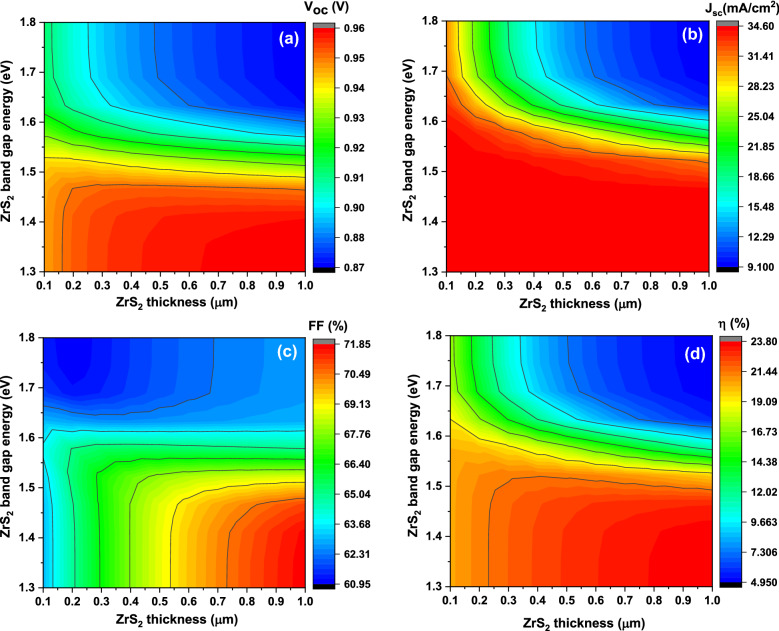
The simulated solar cells basic parameters including V_oc_ (**a**), J_sc_ (**b**), FF (**c**), and η (**d**) as function on the window layer thickness (x-axis) and the band gap (y-axis) at donor concentration 10^19^ cm^−3^.

Figure 6 proves the achieved contour plot of the simulated basic parameters of solar cell as a function on the ZrS_2_ thin film thickness and the band gap vary from 0.1 to 1 μm (the x-axis) and from 1.3 to 1.8 eV (the y-axis), respectively.

The **V**_**oc**_ has different values with different zones as shown in Fig. 6a. It decreases mainly from about 0.96 V to about 0.87 V with band gap increasing from 1.3 to 1.8 eV. For lower band gap from 1.3 eV to about 1.45 eV, the **V**_**oc**_ value improving for each individual **E**_**g**_ value with rising ZrS_2_ thickness. While for intermediate ones from 1.45 eV to about 1.6 eV, the **V**_**oc**_ values remains nearly constant with increasing ZrS_2_ thickness. For higher band gap than 1.6 eV, the **V**_**oc**_ reducing with increasing ZrS_2_ thickness.

Figure 6b appears that the **J**_**sc**_ value remains constant at average of about 33.5 mA/cm^2^ for the **E**_**g**_ value below 1.55 eV even with increasing ZrS_2_ thickness. For the **E**_**g**_ from 1.55 to 1.65 eV, the **J**_**sc**_ value also remains nearly constant with thickness rising with rang from 18 to 25 mA/cm^2^ for each the **E**_**g**_ value. On another hand, for higher **E**_**g**_ values, the **J**_**sc**_ reduces from about 28 mA/cm^2^ to about 10 mA/cm^2^ with thickness increasing from 0.1 to 1 µm.

Figure 6c suggests that the FF has different values with the **E**_**g**_ values. For the **E**_**g**_ below 1.55 eV, FF increasing with ZrS_2_ thickness increasing for each individual value of the **E**_**g**_. For example, at 1.4 eV, FF value increase from about 64% to about 71% with thickness increasing from 0.1 to 1 µm. For intermediate **E**_**g**_ from 1.55 to 1.65 eV, FF remains constant with thickness growing. For higher **E**_**g**_ than 1.65 eV, FF values enhance with thickness increasing but with smaller values than lower band gaps. Overall, the FF value increases mainly with ZrS_2_ thickness increasing.

Figure 6d recognizes that the η has different values where it decreases with thickness increasing for the **E**_**g**_ below 1.5 eV. For example, at 1.4 eV, the η increases from about 20% to about 23.6% with thickness growth from 0.1 to 1 µm. While for intermediate **E**_**g**_ values between 1.55 and 1.65 eV, the η values remains nearly constant with thickness increasing. On another hand, for higher **E**_**g**_, the η reduces with thickness rising for each individual **E**_**g**_ value. Generally, the η has the same shape as the the **V**_**oc**_ values.

The obtained results could be elucidated as the band gap rising, more light path through interface junction and therefore increasing photogenerated rate for the charge carries and then separated and collected easily. At the same time the recombination current as well as the leakage current will be reduced. These factors will enhance the the **V**_**oc**_ value for band gap less than 1.45 eV as previously discussed as in Eq. (3)^37^. On the other hand the **V**_**oc**_ value increase with thickness increasing and therefore improvement of the charge collection length and consequently increasing of electron hole pairs generation through the junction^43^. Moreover, the alignment of bandgaps plays important role to improve the **V**_**oc**_ value where with small band gap for ZrS_2_ than 1.45 eV more confinement take place and therefore the intra-band tunneling for carriers at the interface voltage barrier increases and more current generated^46^. The same description could be applied to explain the behavior of the **J**_**sc**_ values especially for band gap less than 1.55 eV.

By increasing the ZrS_2_ band gap both the **V**_**oc**_ and the **J**_**sc**_ values reduce and this could be attributed to increasing of recombination process as well as the leakage current especially with increasing of ZrS_2_ thickness^2,38^. In such case the mismatch between CuO band gap and ZrS_2_ band gap produce capture centers which increasing the carrier recombination^38^. The behavior of the **FF** could be explained where it is a function on the **V**_**oc**_ values as shown in Eq. (4). The η values mainly followed the sum of the **V**_**oc**_, the **J**_**sc**_, and the **FF** values as presented in Eq. (4)^40^.

**Figure 7:**
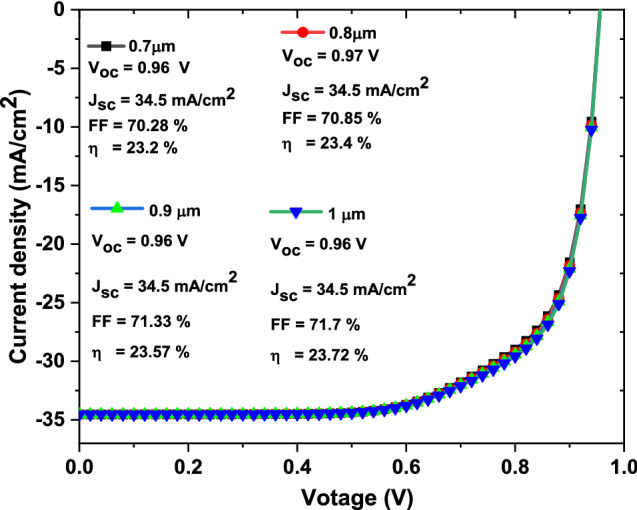
The simulated JV curves with basic parameters of solar cells as function on ZrS_2_ thickness from 0.7 to 1 µm.

From over results we decided that to have higher efficacies devices, the ZrS_2_ thickness and band gap would be in the range from 0.7 to 1 µm and 1.4 eV, respectively. So, JV curves with basic parameters of solar cell devices as function of ZrS_2_ thickness from 0.7 to 1 µm at fixed band gap of 1.4 eV are recorded as shown in Fig. 7. In this case, the **V**_**oc**_ has value from 0.96 to 0.97 V, the **J**_**sc**_ has constant value of 34.5 mA/cm^2^, the **FF** has value from 70.28 to 71.7% and the **η** has value from 23.2 to 23.72% with increasing thickness from 0.7 to 1 µm, respectively.

**Figure 8:**
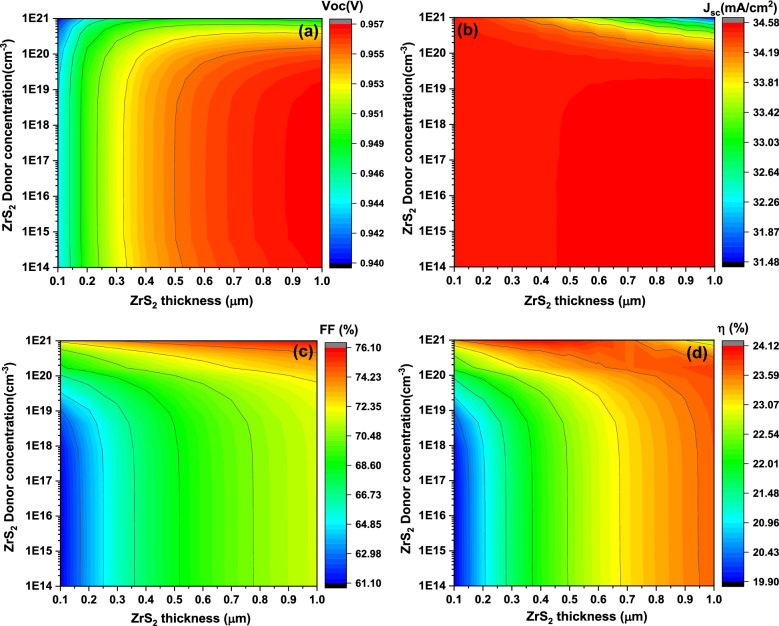
The simulated solar cells basic parameters including V_oc_ (**a**), J_sc_ (**b**), FF (**c**), and η (**d**) as function on the window layer thickness (x-axis) and the carrier concentration (y-axis).

Figure 8 demonstrates the contour plot of the solar cell basic parameters as a function on the ZrS_2_ thin film thickness and the carrier concentration differ from 0.1 to 1 μm (the x-axis) and from 1 × 10^14^ to 1 × 10^21^ cm^−3^ (the y-axis), respectively.

It is evident from Fig. 8a that the **V**_**oc**_ has different sectors depending mainly on the thickness of ZrS_2_. For carrier concentration less than 10^20^ cm^−3^, the **V**_**oc**_ grows from about 0.944 V to about 0.955 V with thickness increasing from 0.1 till 0.5 μm where the **V**_**oc**_ value is almost the same for each thickness. While for higher thickness, the **V**_**oc**_ value remains nearly constant at about 0.957 V. For carrier concentration of 10^20^ and 10^21^ cm^−3^, the **V**_**oc**_ continues almost constant. In general, the **V**_**oc**_ value varies for different carrier concentration and thickness of ZrS_2_ with only small range of about 0.02 V.

The **J**_**sc**_ value stays constant at about 34.5 mA/cm^2^ for all different carrier concentration and thickness of ZrS_2_ as presented in Fig. 8b. Only for carrier concentration of 10^20^ and 10^21^ cm^−3^ and with higher thickness than 0.5 μm, the **J**_**sc**_ decreasing from about 34.5 mA/cm^2^ to 31.5 mA/cm^2^.

Figure 8c implies that the FF increases in sectors from about 61.5 to about 72.5% with thickness increasing and its value remains constant for each thickness with varying of carrier concentration till 10^20^ cm^−3^. While for carrier concentration above 10^20^ cm^−3^, the FF increase from about 70% to about 76%.

Figure 8d realizes that the η has different values with different sectors where it increases mainly from about 20% to about 23.5% with thickness increasing from 0.1 to 1 µm especially for carrier concentration less 10^20^ cm^−3^ and remains constant for each thickness even with different carrier concentration. There is a curved path in which the η has the highest value from about 23.5% to 23.8%. This path located between thickness of 1 µm and from carrier concentration of 10^14^ cm^−3^ to carrier 10^20^ cm^−3^, then for higher carrier concentration above10^20^ cm^−3^ and with ZrS_2_ thickness in the range from 0.3 to 1 µm.

It is clear that the increasing of donor concentrations of ZrS_2_ thin film has no influence on the **V**_**oc**_ value especially with lower than 10^20^ cm^−3^ with each individual thickness. This may be due to full formation of the depletion width (**W**_**d**_) which affected on the generation rate of charge carriers and the diffusion length of minority carriers (holes) in n-ZrS_2_ and (electrons) in p-CuO^2^. So, the photogenerated current is completely related to depletion **W**_**d**_ and therefore full built-in voltage (**V**_**bi**_) which mainly constructed on the side of the p-CuO thin film^37^. On the other hand, the **V**_**oc**_ value increase with increasing of ZrS_2_ thickness for each individual carrier concentration that could be attributed to increase the life time and length of charge collection leading more photogenerated current, therefore leakage current and recombination rate will decline with growing of thin film thickness^45^.

Such suggestions are validated since the increasing of carrier concentration above 10^20^ cm^−3^ produce almost constant value of the **V**_**oc**_ even with thickness increasing. Moreover, the constant value of the **J**_**sc**_ with increasing of both thickness and carrier concentration of ZrS_2_ thin films supported such explanations. Also, the constant** J**_**sc**_ value due to that the window layer does not perform a huge part in the solar spectrum absorbing where it is mainly managed as conducting path for electrons^47^.

**Figure 9:**
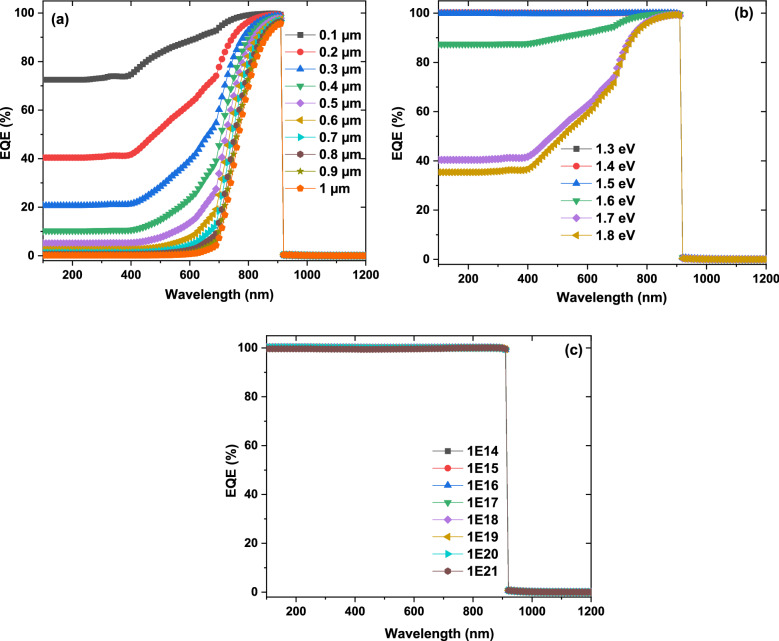
The simulated external quantum efficiency as function on ZrS_2_ thickness (**a**), band gap (**b**), and the carrier concentration (**c**), respectively.

The η values reflect the total behavior of the **V**_**oc**_, the **J**_**sc**_, and **FF** where its value mainly follows the **V**_**oc**_, and **FF**. The photogenerated current is investigated where EQE is simulated as function on ZrS_2_ thickness, band gap, and the carrier concentration as stated in Fig. 9. It is certain that EQE value reduce from about 72% to around zero with increasing thickness from 0.1 to 1 µm at higher energy and from 100% to around 80% at lower energy. With raising of the band gap, the EQE value downward from 100 to 38% especially for higher wavelength. This reducing for EQE with increasing of thickness and band gap could be attributed to improving of the recombination rate and decreasing the charge carrier collection and therefore decline of the separation of the generated current^46^. The EQE has the same value and shape with different carrier concentration. EQE results are agreed and supported the obtained values of **J**_**sc**_ for ZrS_2_ thin thilm thickness, band gap, and carrier concentrations.

**Figure 10:**
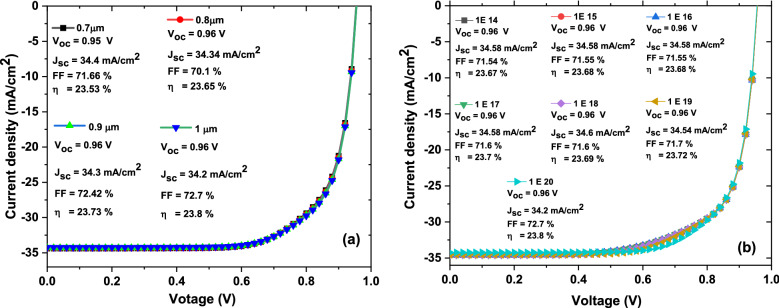
The simulated JV curves with basic parameters of solar cells as function on ZrS_2_ thickness (**a**) and carrier concentration (**b**).

Figure 10 describes the obtained JV curves with solar cell basic parameters with variation of ZrS_2_ thin film thickness and carrier concentration. From Fig. 10a, the **η** value increase from 23.53 to 23.8% with thickness increasing from 0.7 to 1 µm, while the carrier concentration for ZrS_2_ thin film is fixed at above10^20^ cm^−3^. On the other hand, from Fig. 10a and at constant ZrS_2_ thickness of 1 µm So, the **η** value increase from 23.67 to 23.8% with increasing of carrier concentration from 10^14^ to 10^20^ cm^−3^. Additionally, our simulations results indicate that to have higher efficiencies of inorganic ZrS_2_/CuO heterojunction solar cell devices, the CuO band gap, thickness, and carrier concentration would be about 1.35 eV, 5.5 µm, and above 10^18^ cm^−3^. While, the ZrS_2_ band gap, thickness, and carrier concentration would be about 1.4 eV, 1 µm, and less than 10^20^ cm^−3^ where the highest efficiency of about 23.8% was achieved with the **V**_**oc**_ value of 0.96 V, the **J**_**sc**_ value of 34.2 mA/cm^2^, and the **FF** value of 72.2%. Our results indicate that ZrS_2_/CuO solar cell devices could be comparable with one based on In_2_S_3_. The optimum devices of Cu(In, Ga)Se_2_, CuIn(S,Se)_2_ and Cu_2_ZnSnS_4_ based solar cells, where In_2_S_3_ was the buffer layer (50–125 nm thickness), fluorine-doped tin oxide was window layer and gold (Au) was used for back contact, revealed highest efficiencies in the range of 16.94–22.50%^49^. Additionally, Reyes et al. developed FTO/TiO_2_/MASnI_3_/Cu_2_ZnSnS_4_/Au as a *n-i-p* heterojunction perovskite solar cell^48^. They reported that when the acceptor and defect densities were 10^16^ cm^–3^ and 10^14^ cm^–3^, respectively, the best values (**V**_oc_ of 0.96 V, **J**_sc_ of 31.66 mA/cm^2^, FF of 67% and **η** of 20.28%), were obtained^50^.

### Impact of operation temperature on JV curves and the basic parameters of optimum ZrS_2_/CuO solar cell

**Figure 11:**
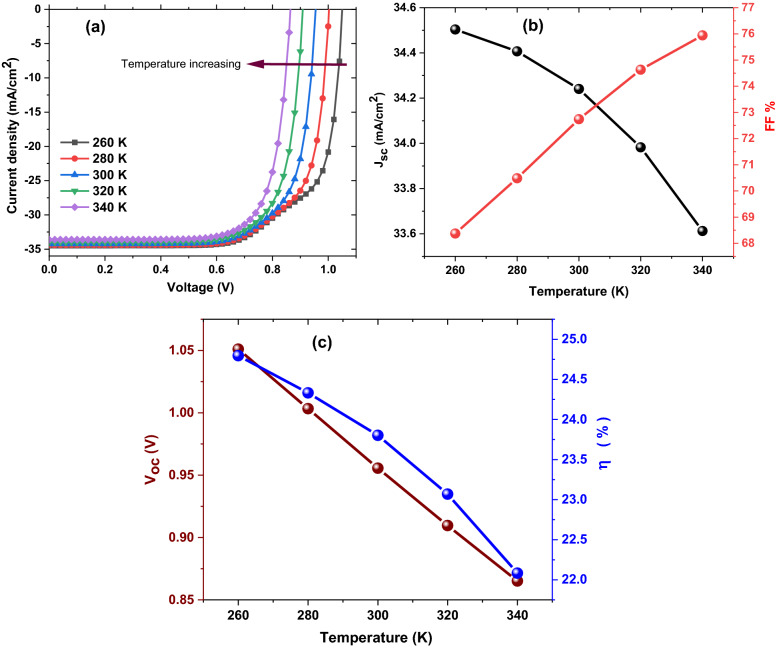
The simulated JV curves and the basic parameters of optimum ZrS_2_/CuO solar cell as function on operation temperature (K).

In this section, the influence of operation temperature on JV curves and the basic parameters of the optimum ZrS_2_/CuO solar cell was investigated as presented in Fig. 11. It is apparent that increasing of operation temperature minimize the **V**_**oc**_ and the **J**_**sc**_ and therefore the η where their values decreasing from 1.05 to 0.87 V, 34.5 to 33.6 mA/cm^2^, and 24.8 to 22.1%, respectively with rising temperature from 260 to 340 K.

Such results could be explained employing Eq. (3) where the reverse saturation current (**I**_**o**_) increases by temperature increasing as well as the growing of the recombination probability for the charge carriers, and therefore the **V**_**oc**_, the **J**_**sc,**_ and the η values reduce^44^. The results indicate small variation in the η value especially with the average temperature all over the world and consequently the applicable of ZrS_2_/CuO solar cell to fabricate higher performance photovoltaic devices.

**Figure 12:**
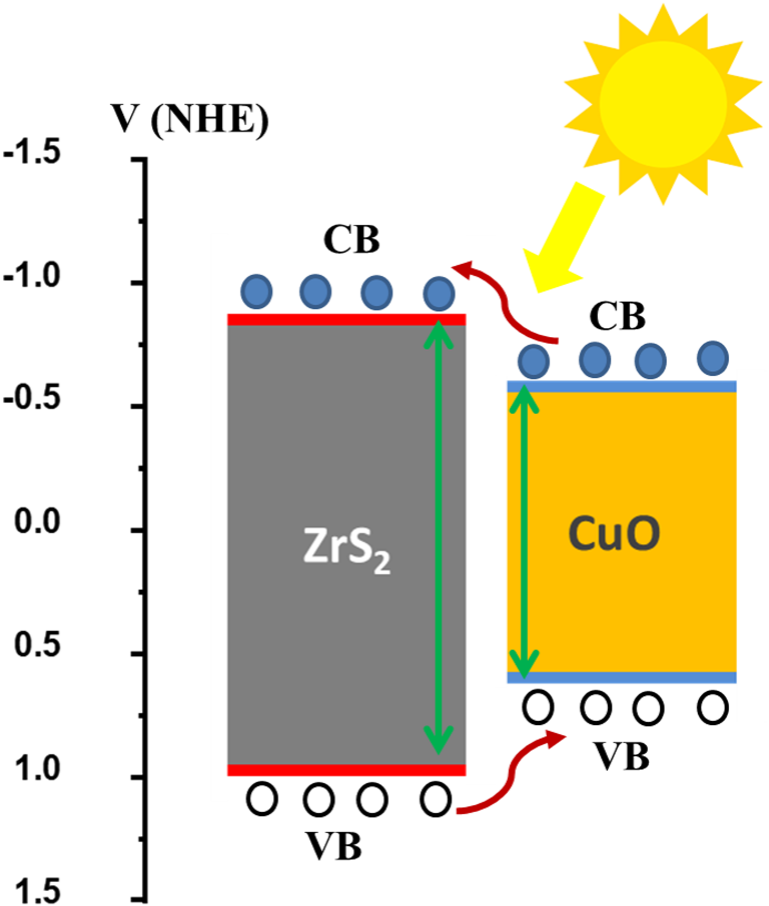
The schematic for the energy band diagram of inorganic ZrS_2_/CuO heterojunction.

The explanations for the effect of carrier concentration and bandgap on the fundamental parameters of inorganic ZrS_2_/CuO heterojunction solar cells could be cleared by the energy band diagram for the window and absorber layers as presented in Fig. 12^51,52^. The figure indicates that the valence band (VB) and conduction band (CB) energies of ZrS_2_ and CuO thin films are with good match together which increasing of the generation and separation rates of generated the electron‐hole pairs. In such case, when the heterojunction absorbing photons, the electron‐hole pairs generate and therefore electrons move from CB of CuO thin film to CB of ZrS_2_ thin film while holes move from VB of ZrS_2_ thin film to VB of CuO thin film then they are separated at the front and back contacts. By increasing the carrier concentration, the generation of electron‐hole pairs will increase as well as the photocurrent. Moreover, when the band gaps values are 1.35 and 1.4 eV for ZrS_2_ and CuO thin films, the alignment of band energies will increase and therefore the efficiency will greatly be enhanced.

## Conclusions

In conclusion, novel inorganic ZrS_2_/CuO heterojunction solar cells was simulated for the first time using SCAPS-1D software. After successfully simulation process, the optimum thickness, carrier concentration, and bandgap for CuO thin film were found to be 1.35 eV, 5.5 µm, and above 10^18^ cm^−3^, respectively, and for ZrS_2_ thin film to be 1.4 eV, 1 µm, and less than 10^20^ cm^−3^, respectively to have higher solar cell efficiency. Employing such parameters, the highest **η** of 23.8% was realized with the **V**_**oc**_ value of 0.96 V, the **J**_**sc**_ value of 34.2 mA/cm^2^, and the **FF** value of 72.2%. The photogenerated current, the recombination rate, the collection charge carrier length, the minority carrier lifetime, width of the depletion layer, the built-in potential are the elements that manipulated the performance of solar cell basic parameters. Application of our simulation outcomes on the experimental area could help to obtain high-efficiency photovoltaic devices based on ZrS_2_/CuO heterojunctions.

## Data Availability

The datasets used and/or analysed during the current study available from the corresponding author on reasonable request.
